# Therapeutic and Surgical Indications for Patients with Penile Cancer in the COVID-19 era

**DOI:** 10.1590/S1677-5538.IBJU.2020.S110

**Published:** 2020-07-27

**Authors:** Nelson Canales Casco, María Jiménez Carmona, Álvaro Juárez Soto

**Affiliations:** 1 Hospital de Jerez de la Frontera Cádiz Spain Hospital de Jerez de la Frontera, Cádiz, Spain

**Keywords:** Penile Neoplasms, Clinical Protocols, COVID-19 [Supplementary Concept], Pandemics

## Abstract

**Purpose:**

The aim of this work is to review and synthesize the existing evidence and recommendations regarding to the therapeutic and surgical indications as well as monitoring of patients with Penile Cancer in COVID-19 era and to propose an action protocol to facilitate decision-making.

**Material and Methods:**

A non-systematic review of the literature regarding the management of penile cancer during the COVID-19 pandemic was performed until April 30, 2020. We propose our recommendations based on this evidence.

**Results:**

Penile cancer is an uncommon but aggressive disease. Prognosis is determined by several characteristics, being the most important the presence of lymph nodes, in which case, treatment should not be delayed. For these reasons, an initial evaluation is mandatory. Priority classifications, based on the oncological outcomes when treatment is delayed, have been made in order to separate deferrable disease from the one that needs high priority treatment. In penile cancer with low risk of progression, surgical treatment can be delayed, but other options must be considered, like topical treatment or laser therapy. In cases with intermediate risk of progression, surgical treatment may be delayed up to three months, but we must consider radiation therapy and brachytherapy as effective options. When feasible, follow-up should by telemonitoring.

**Conclusions:**

In the COVID 19 era, initial evaluation of the patient is mandatory. Histological diagnosis with local staging is necessary before offering any therapeutic option. In case of superficial non-invasive disease, topical treatment is effective in absence of lymph node involvement. In selected patients, radiotherapy is an organpreserving approach with good results. Non-deferrable surgical treatment must be performed by an experienced surgeon and as an outpatient procedure when possible. When indicated, iLND should not be delayed since it is decisive for patient survival. Follow-up should be by telemonitoring.

## INTRODUCTION

Penile cancer is an uncommon pathology, with an overall incidence, in industrialized countries, of around 1/100.000 males in Europe and USA (1). The incidence is affected by race and ethnicity, with the highest incidence in white Hispanics, followed by Alaskans and native American Indians. In other parts of the World, such as South America, South East Asia and parts of Africa, the incidence is much higher (2). Several risk factors have been identified, such as HVP infections, smoking, phimosis, chronic penile inflammation and multiple sexual partners (3, 4).

Winters et al. made an analysis of the United States national cancer database from 1998 to 2012, describing that the presence of pathological subtype of the disease, perineural and lymphovascular invasion, depth of the invasion and grade in the primary tumor will determine the prognosis (5).

When analyzing the social characteristics of the patients, Jimenez Ríos et al. described that low socioeconomic status, poor education and delay in seeking medical attention are related with advanced disease. This analysis was made in Mexico and concluded that a delay of 10 months between the appearance of the first symptoms and the patient seeking for medical attention was related with advanced disease and worse prognosis (6).

On March 11, 2020, the World Health Organization declared COVID-19 a global pandemic (7). This has led to dramatically changes on medical and surgical priorities. Postponement for all outpatient and elective activities to save facilities and resources for urgent cases and COVID-19 patients have been adopted by most hospitals in the affected countries. We know that cancer patients are characterized by their higher susceptibility to infectious diseases compared to general population with 3.5 folds increased risk of COVID-19 related serious events (8). Therefore, the choice of urgent and emergent surgeries that should still occur will depend on the capacity and demand, but also must be counterbalanced by the effects of delaying surgeries (9). As COVID-19 continues to spread, governments have imposed increasingly aggressive measures that have demonstrated benefits in reducing the spread of the virus minimizing the impact that cases have on local health care systems (7). This is generating a rapid and tragic health emergency worldwide, due to the need to provide assistance to an overwhelming number of infected patients and, at the same time, treat all the non-deferrable oncological and benign conditions (10).

Liang W et al. in a nationwide analysis of cancer patients with COVID-19 infection proposed that intentional postponing of adjuvant chemotherapy or elective surgery for stable cancer should be considered in endemic areas (11).

Later, Xia Y et al. reported that these findings can't be generalizable due to the small sample analyzed, the heterogeneity on the type of cancers, the great difference in the course of the disease and the different treatment strategies. (12) Uncareful delay of onco-urologic surgeries may have an impact on short-term progression and/or mortality (8).

Knowing that penile cancer is an uncommon, but yet aggressive disease, there is a need to have easy to follow strategies and protocols for early diagnosis, adequate treatment and follow up during COVID-19 pandemic.

## MATERIAL AND METHODS

A non-systematic review of the available literature on the management of penile cancer during the COVID-19 pandemic was performed. We navigated through Pubmed, Cochrane library, the American Urology Confederation (CAU) library of COVID-19, we reviewed the European Urology Association (EUA) Rapid Reaction Group recommendations, the British Association of Urological Surgeons (BAUS) recommendations, the National Comprehensive Cancer Network (NCCN) guidelines and the American Urology Association (AUA) COVID-19 library in search of literature available in English and Spanish until April 30, 2020.

We described and analyzed the different protocols and recommendations proposed by different authors and scientific organizations, and based on this evidence and our experience, we created an action protocol for early and safe diagnosis, adequate treatment and follow up of patients with penile cancer during COVID-19 pandemic.

## RESULTS

COVID-19 pandemic has led to dramatically changes on medical and surgical priorities. Postponement for all outpatient and elective activities to save facilities and resources for urgent cases and COVID-19 patients have been adopted by most hospitals in the affected countries (8). This is a problem that is affecting health services worldwide.

Naspro and Da Pozzo stated that the real challenge in this time, is that the health service in Italy is currently unable to easily deal with other conditions except treatment of COVID-19. Urologists manage patients with oncological diseases and surgical priorities, as well as non-oncological conditions that can affect quality of life. Regional guidelines require that any patient requiring oncological surgery must be treated within 30 days from diagnosis. Departments struggle to meet this deadline normally, now it's even harder (13).

Pulliati et al.(8) in a review of the literature available of COVID-19 and Urology, quotes the work of Liang et al. (11), who stated that cancer patients are characterized by their higher susceptibility to infectious disease compared to general population with 3.5 folds increased risk of COVID-19 related serious events in the form of intensive care admission, requirement for mechanical ventilation, or death due to their immune compromised state related to the nature of their malignancy and the anti-cancer management (chemotherapy, radiotherapy, or surgery). This paper recommends to delay all elective cancer surgeries or adjuvant chemotherapy in patients with stable cancer. Wang et al. (14) reported that the major risk factor for cancer patients during COVID-19 pandemic is the inability to receive sufficient medical support.

Xia Y et al. (12) reported that Liang et al. (11) findings are not generalizable due to the small sample analyzed, the heterogeneity on the type of cancers, differences in their course and the different treatment strategies.

In this same line, Méjean A. et al. as part of the Cancerology Committee of the French Association of Urology (CCAFU) published expert opinion recommendations based on literature review for cancer treatment (15). They stated that a 3-month delay in diagnose and treatment of penile cancer decreases the possibility of conservative treatment. This 3-month delay seems to have no impact in 5-year overall survival or recurrence free survival. After 6-month delay, survival reduces after 2 years. A 3-month delay in the treatment of lymph nodes, significantly decreases 5-year specific survival (39.5% when compared with 64.1% when there is no delay). This idea reinforces the statement of Xia et al. that we cannot treat all patients and all pathologies as a same (12).

We know that prognosis is determined by the pathological subtype of the disease. In this case, the verrucous subtype is considered to demonstrate low malignant potential, while adenosquamous and sarcomatoid variants carry a worse prognosis. The presence of perineural and lymphovascular invasion, depth of the invasion and grade in the primary tumor are also relevant in determining prognosis (2, 16).

This is the reason why, potential new penile cancers require clinical assessment, as stated by the British Association of Urological Surgeons (BAUS) (17), in order to determine the most appropriate treatment. It is vital to perform a good physical evaluation of the penile lesion (s) with all its characteristics (diameter, location, number, morphology and involvement of other structures) as well as the presence of suspicious lymph nodes. To complete this initial evaluation, histologic diagnosis with punch, incisional or excisional biopsy is paramount in determining the treatment algorithm (2, 16).

Once histological diagnosis has been made, standard treatment for superficial non-invasive lesions; Tis or Ta should be with penis-preserving techniques including topical treatments, laser therapy, Mohs surgery and conservative penile surgery. In T1G1-2 disease, careful consideration should be given for penile-preserving techniques if the patient is reliable in terms of complications with close follow-up. This includes wide local excision, glansectomy in selected cases, Mohs surgery, laser therapy and radiation therapy. In T1G3 or ≥T2 extensive surgery, RT and in some cases, brachytherapy may be feasible (2, 16).

The presence and extent of regional inguinal lymph node metastases have been identified as the single most important prognostic indicator in determining long-term survival (16).

During COVID-19 pandemic, there's a need to create easy to follow recommendations and protocols that are adapted to the actual health situation in order to guarantee a safety environment for the patient and medical staff. That's why different associations, expert groups and authors are doing great efforts to review available data and provide adapted strategies.

There's a need to classify oncological diseases in groups according to their clinical stage, and state priorities for treatment.

The European Urology Association (2), through the guidelines office, proposed a rapid reaction group (GORRG) on 19th March 2020, to facilitate the development of adapted guidelines to deal with a range of situations and priorities. Levels of prioritization were established taking in count the impact of delay on primary outcomes, possibility of alternative methods that could replace the standard procedure with less operating room requirements, presence of co-morbidities and/or increased risk of complications among others. Low priority was used to classify diseases that were very unlikely to cause clinical harm if postponed 6 months. Intermediate priority for diseases that can cause clinical harm if postponed 3-4 months even though it's unlikely. High priority when disease can cause clinical harm if postponed more than 6 weeks, and emergency for life threatening situations. A similar classification was made by Ficarra V. et al. who distinguished urological procedures to treat cancer in four categories: a) Non deferrable, b) Semi-non-deferrable, c) Deferrable, d) Replaceable by another treatment (10).

Goldman et al. from Cleveland Clinic urology department made a tier system from 0-4 to help prioritize surgical procedures. In this classification they used five priority tiers, where 0 is considered an emergency, and 4 a non-essential surgery that can be delayed for long time. Penile cancer is listed a priority 1 in this tier classification. This classification was based on the available data regarding risk of progression but mostly from expert opinion (18).

In this case we made our classification (Table-1) based on the classification system proposed by Ficarra V. et al. which is similar as the proposed by the EUA (2), because it allows us to stratify one same disease in a different category depending on its clinical stage. The time of delay in deferrable disease will be 6 months and in semi non-deferrable 3 months (10).

**Table 1:** Treatment priority classification for Penile Cance during COVID-19 pandemic

Penile Cancer Priority Category	Non-Deferrable	Semi non - Deferrable	Deferrable	Emergency
	(6 weeks)	(up to 3 months)	(6 months)	
Definition	≥ T1G3, any N+	T1G1, T1G2 (non verrucous)	Tis, Ta and some T1G1	Life-threatening situations, opiod-depending pain, urinary flow obstruction.

Adapted from Ficarra et al. and the EUA GORRG recommendations (2, 10)

The presence of inguinal lymph nodes will automatically classify the disease as non-deferrable. In semi-deferrable disease, the possibility to access priority medical assistance or telemonitoring should be available. During this time, the patient must perform self-examination and in case of noticing palpable inguinal lymph nodes, medical attention should be a priority.

In this case, in order to complete diagnosis, chest, abdomen and pelvic CT scan should be performed (2, 16, 19).

In a literature review made by Wallis C. et al. they describe that patients who receive early inguinal lymphadenectomy; with median time to surgery of 1.7 months, range 0-6 months, had significantly lower five-year cancer specific mortality than those who underwent delayed intervention (20).

In deferrable disease, when other treatment options are available, they must be offered.

Méjean A. et al. (15) proposed that when all therapeutic options are available and low risk disease is being treated; Tis (PeIN), topical treatment should be the first option. In case of conservative treatment failure, if strict supervision is available (this may be done by telemonitoring), reasonable delay on surgery may be an option, and, when invasive disease; <T1G2 or verrucous cancer, with low risk of lymph node affection, a delay on surgery must be discussed with the need of lymph node evaluation. Alchiede Simonato et al. (19) made a simplification of the diagnostic therapeutic algorithms based on the EUA guidelines and the experience gained in Italy during this crisis. They proposed that, when indicated, penectomy with inguinal lymph node dissection must not be delayed. We understand that this refers to patients with ≥T1G3, or any N+. This was also stated by Puliati et al. (8).

Following the same line, Stensland et al. (9), suggested a triage for surgical cancer treatments, where they recommend to not delay treatment of possible clinically invasive penile cancer or obstructive cancers. Prevention of lymph node metastases is vital to spare significant patient morbidity.

They emphasized the importance of performing surgery as an outpatient procedure when feasible.

**Figure 1:**
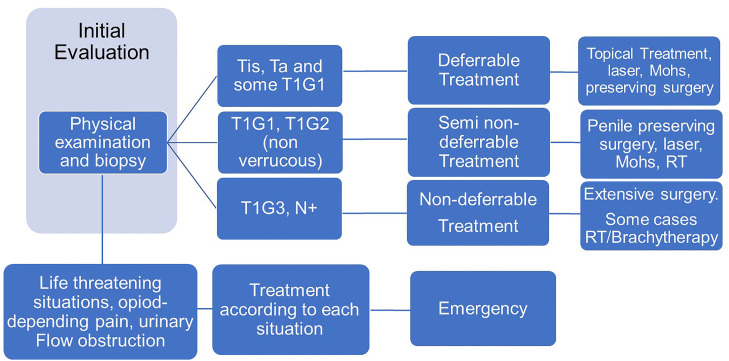
Decision-making recommendations for Penile Cancer during COVID-19 era.

In order to facilitate decision-making for treating penile cancer during COVID Era, we created a recommendation flowchart (Figure-1).

Simonato A. et al. (19), the EUA protocol created by the GORRG (2) and the BAUS recommendations (17) all coincide that follow-up should be by remote monitoring.

## DISCUSSION

We made a review of the literature available at the moment, for the diagnosis, treatment and follow-up of penile cancer during COVID-19 pandemic. Despite the lack of information at this point, several authors and scientific organizations are moving quickly to make different protocols and guides of recommendations to facilitate decision--making in urological cancer disease during the COVID-19 pandemic.

Based on the characteristics of penile cancer; epidemiological, histological and clinical behavior, we know we are dealing with an uncommon but aggressive disease, with high mortality when diagnosis and treatment are delayed. During COVID-19 pandemic hospitals worldwide have been forced to make changes in order to save facilities and resources for urgent cases. This has led to postponement of outpatient and elective activities.

As more information appears, protocols are being adapted. Liang et al. (11) recommended to delay all elective cancer surgeries or adjuvant chemo-therapy in patients with stable cancer. This was based on the premise that oncological patients have higher susceptibility to infectious diseases. It's well known that evolution, treatment response and prognosis vary between different cancer diseases, so we must treat each one individually, as reported by Xia et al. (12) and Wang et al. (14).

Different classifications have been made in an attempt to establish levels of prioritization. The EAU guidelines (2), Ficarra et al. (10), Méjean et al. (15), the BAUS guidelines (17) and Goldman et al. (18) agree that different cancer diseases can be classified in low priority (deferrable), intermediate priority (semi non-deferrable), high priority (non-deferrable) and emergencies, depending on the probable onco-logical outcome if attention is delayed. In terms of treatment the EAU guidelines (2), Stensland et al. (9), Ficarra V. et al. (10), Méjean et al. (15), the NCCN Guidelines (16), Simonato (19) and Wallis (20) all recommend special attention to the presence of inguinal lymph nodes because they will affect prognosis. In case of affection, treatment must not be delayed.

During COVID-19 pandemic, authors agree that, when feasible, follow-up can be done by remote monitoring, phone calls or even by sending pictures (2, 17, 19).

## CONCLUSIONS

In the COVID era, evaluation of the patient is necessary in order to clinically determine the stage of the disease and proceed to adequate treatment. Appropriate safety measures must be taken to guarantee the safety of the patient and medical staff. Histological diagnosis with local staging must be obtained before offering a therapeutic option. In case of superficial non-invasive disease, topical treatment is effective and should be the first option in absence of lymph node involvement. In selected patients with T1-2 lesions <4cms in diameter, radiotherapy is an organ-preserving approach with good results. It can be given as external radiotherapy combined with brachytherapy boost or as brachytherapy alone.

When surgical treatment can't be delayed, it should be performed by an experienced surgeon, under appropriate safety measures and as an outpatient procedure when feasible. When indicated, LND should not be delayed since it is decisive for patient survival. The follow up should be performed by telemonitoring when possible.

## ABREVIATIONS

CAU: American Urology Confederation
EAU: European Urology Association
GORRG: Guidelines Office Comissioned a Rapid Reaction Group
NCCN: National Comprehensive Cancer Network
BAUS: British Association of Urological Surgeons
